# Transcriptional Regulators in the Cerebellum in Chronic Schizophrenia: Novel Possible Targets for Pharmacological Interventions

**DOI:** 10.3390/ijms26083653

**Published:** 2025-04-12

**Authors:** América Vera-Montecinos, Belén Ramos

**Affiliations:** 1 Psiquiatria Molecular, Parc Sanitari Sant Joan de Déu, Institut de Recerca Sant Joan de Déu, Dr. Antoni Pujadas 42, 08830 Sant Boi de Llobregat, Spain; america.vera@uss.cl; 2 Departamento de Ciencias Biológicas y Químicas, Facultad De Ciencias, Universidad San Sebastián, Sede Tres Pascualas Lientur 1457, Concepción 4080871, Chile; 3 Centro de Investigación Biomédica en Red de Salud Mental, CIBERSAM (Biomedical Network Research Center of Mental Health), Ministry of Economy, Industry and Competitiveness Institute of Health Carlos III, 28029 Madrid, Spain; 4 Faculty of Medicine, University of Vic-Central University of Catalonia, 08500 Vic, Spain

**Keywords:** schizophrenia, cerebellum, transcription factors

## Abstract

Despite the emerging evidence of the role of transcriptional regulators in schizophrenia as key molecular effectors responsible for the dysregulation of multiple biological processes, limited information is available for brain areas that control higher cognitive functions, such as the cerebellum. To identify transcription factors that could control a wide panel of altered proteins in the cerebellar cortex in schizophrenia, we analyzed a dataset obtained using one-shot liquid chromatography–tandem mass spectrometry on the postmortem human cerebellar cortex in chronic schizophrenia (PXD024937 identifier in the ProteomeXchange repository). Our analysis revealed a panel of 11 enriched transcription factors (SP1, KLF7, SP4, EGR1, HNF4A, CTCF, GABPA, NRF1, NFYA, YY1, and MEF2A) that could be controlling 250 altered proteins. The top three significantly enriched transcription factors were SP1, YY1, and EGR1, and the transcription factors with the largest number of targets were SP1, KLF7, and SP4 which belong to the Krüppel superfamily. An enrichment in vesicle-mediated transport was found for SP1, KLF7, EGR1, HNF4A, CTCF, and MEF2A targets, while pathways related to signaling, inflammation/immune responses, apoptosis, and energy were found for SP1 and KLF7 targets. EGR1 targets were enriched in RNA processing, and GABPA and YY1 targets were mainly involved in organelle organization and assembly. This study provides a reduced panel of transcriptional regulators that could impact multiple pathways through the control of a number of targets in the cerebellum in chronic schizophrenia. These findings suggest that this panel of transcription factors could represent key targets for pharmacological interventions in schizophrenia.

## 1. Introduction

Schizophrenia (SZ) is a polygenetic psychiatric disorder with heritability of up to 80% [1]. The mechanisms underlying this disorder are complex and are not completely understood. However, hypotheses such as neurodevelopmental and cognitive dysmetria have been proposed as a framework for the understanding of this psychiatric disorder. The neurodevelopment hypothesis argues that genetic predisposition and possible alterations during intrauterine life could lead to the altered development of the central nervous system (CNS), which could manifest during adolescence [2,3,4]. In recent decades, it has been suggested that the cerebellum is implicated in this pathophysiology through the cognitive dysmetria hypothesis [5]. This hypothesis states that dysfunction of the cortico-thalamo-cerebellar circuit (CCTC) contributes to symptom emergence in SZ [6,7,8]. In the context of CCTC, the cerebellum innervates through the thalamus to the prefrontal and parietal cortex, areas involved in cognitive functions and altered in SZ [9]. The cerebellum is a highly organized tissue, consisting of a homogeneous neuronal population, with granular cells making up approximately 90% [10]. This feature makes the cerebellum a useful model for proteomic study, allowing us to find molecular alterations that could alter internal circuits.

Transcription factors (TFs) control gene networks that are required for the processes of regionalization and neuronal precursor migrations during cerebellar development [11]. In the context of SZ, it is known that several signaling pathways are dysregulated; therefore, it is necessary to identify the transcriptional programs that regulate the differentially expressed genes involved in the altered pathways. In this context, studies have associated the altered expression of several TFs such as *TCF4* with a high risk of SZ [12]. This relationship could be likely explained by the fact that during development, TCF4 is essential for neuronal migration during cortex cerebellar development [13]. Also, it is known that dendritic organization could be affected in SZ. The altered expression in the postmortem cerebellum of some members of the SP/KLF protein superfamily, known as Specificity Proteins (SPs), has been related to altered dendritic organization and neuronal growth in SZ [14,15], as well as Krüppel-like factors (KLFs) in neuronal morphogenesis [16,17]. In addition, the transcriptional dysregulation of NKX2-1 and EGR1 has been correlated with altered GABAergic neurotransmission in SZ [18], which could lead to altered synaptic processes and the poor cognitive function described in SZ. Thus, the accumulative effect of the altered expression of these TFs could cause the dysregulation of transcriptional networks, which could compromise the neuronal structure and synaptic efficiency and lead to the dysfunction of signaling pathways seen in SZ. However, the identification of transcriptional factors that could modulate large networks of altered genes in the cerebellum in SZ and how these transcription factors impact specific pathways and biological functions has not yet been studied in depth.

Our aim was to identify possible transcriptional regulators in the cerebellum that could be responsible for altered levels of different proteins. In addition, we further investigated the biological processes and signaling pathways controlled by transcription factor-dependent altered programs.

## 2. Results

We analyzed a previous dataset of 250 altered proteins in the human cerebellum cortex in chronic SZ, obtained from a proteomic study using one-shot liquid chromatography–tandem mass spectrometry [19] (see Table S1 for more details). The dataset for the proteomic profile of the cerebellum was deposited in the ProteomeXchange repository with PXD024937 as an identifier.

**Figure 1:**
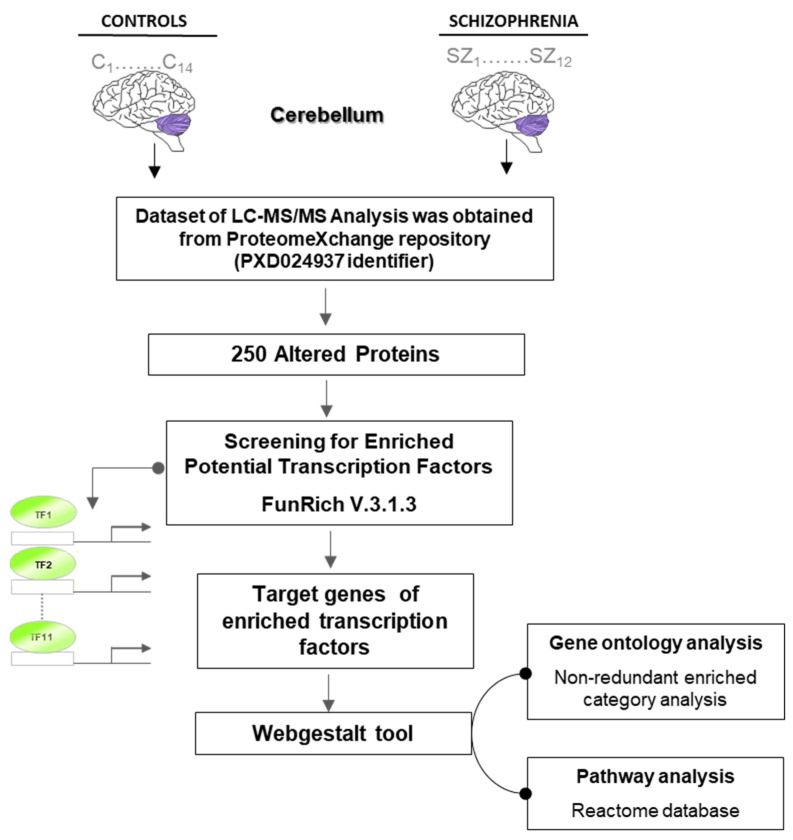
Experimental design to identify enriched transcription factors and their dependently altered biological processes and pathways in the cerebellum in schizophrenia.

To carry out the study, we performed an experimental design, shown in Figure 1, where the 250 altered proteins were used to search for transcription factors that could be controlling them. To find the biological processes and pathways that could be regulating these transcription factors, we performed gene ontology analysis with the protein groups regulated by each transcription factor.

### 2.1. Putative Transcriptional Programs Responsible for Changes in the Proteomic Profile in the Cerebellum

**Figure 2:**
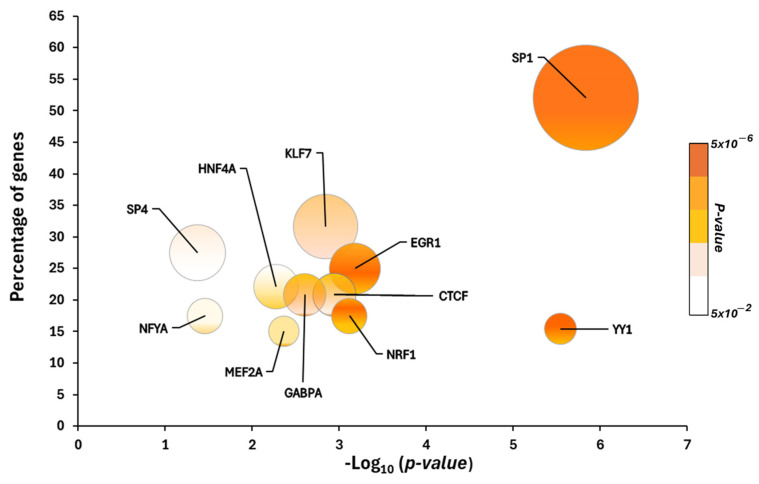
Potential transcription factors involved in the regulation of the altered proteins in the cerebellum of chronic schizophrenia patients. The *X*-axes show the −log_10_ enrichment *p*-value. The *Y*-axes show the percentage of target genes for each transcription factor. The size of each bubble indicates the number of protein targets.

To investigate the transcriptional program that could control the 250 altered proteins in SZ, we performed an enrichment analysis on TFs. Our enrichment analysis for the transcription factor targets showed 40 significant TFs (*p*-value < 0.05) (Supplementary Dataset S1). We generated a list of 11 potential TFs that could be controlling the 250 altered proteins according to the following criteria: the TFs would regulate more than 15% of the target proteins (Figure 2). These TFs were SP1, KLF7, SP4, EGR1, HNF4A, CTCF, GABPA, NRF1, NFYA, YY1, and MEF2A. This analysis revealed that the top three most significant TFs were SP1, EGR1, and YY1, with 125, 60, and 37 targets, respectively (Supplementary Dataset S2). Furthermore, the analysis showed that the TFs with the largest percentage of target proteins were SP1 (125 targets), KLF7 (76 targets), and SP4 (66 targets), all of which belong to the Krüppel superfamily.

### 2.2. Altered Biological Processes Controlled by Transcriptional Programs in the Cerebellum in Chronic Schizophrenia

**Figure 3:**
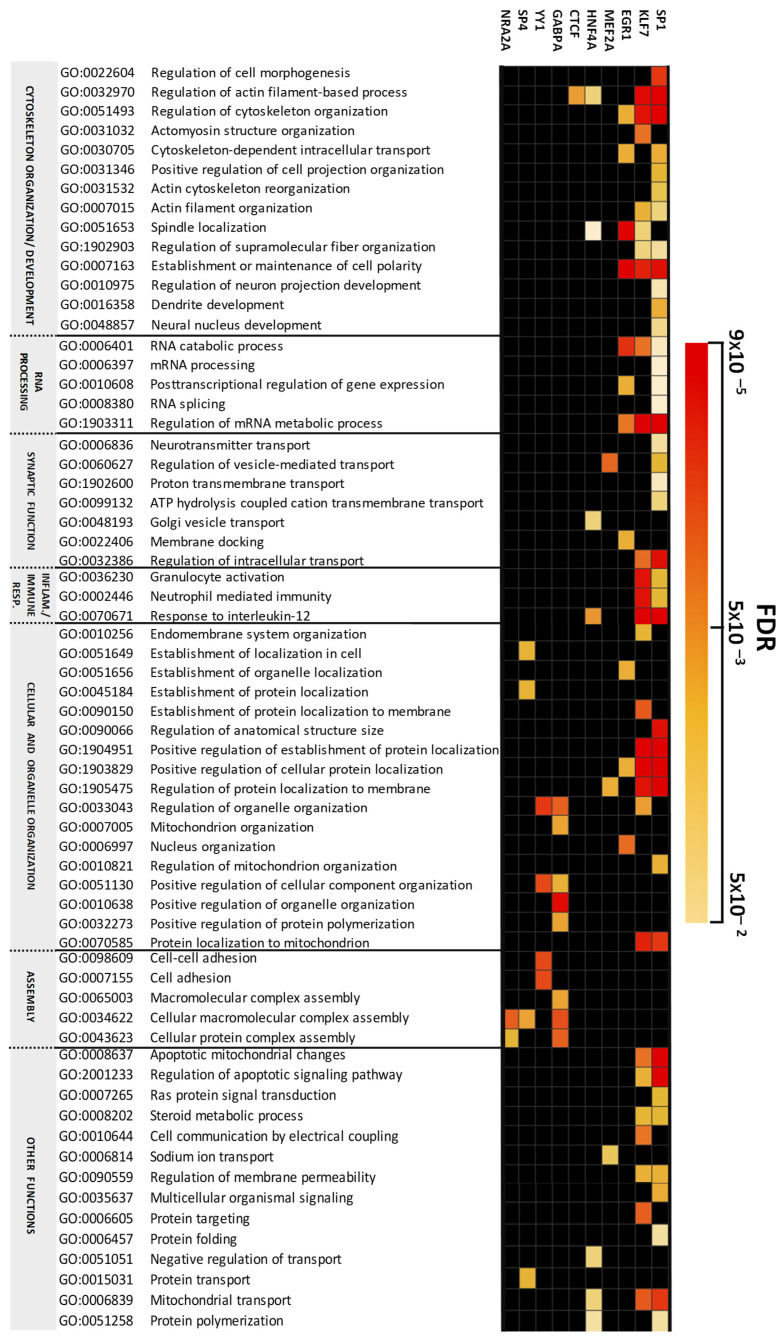
Non-redundant enriched biological process categories for altered targets of transcription factors. The enrichment analysis was performed using Webgestalt, and the heat map visualization of enriched biological process was created using Perseus software.

Our gene ontology analysis of target genes revealed that 10 out of 11 TFs had enriched biological processes (FDR < 0.05). The most significant biological processes were regulated by SP1, KLF7, EGR1, and GABPA (Figure 3). In this analysis, the SP1 and KLF7 target proteins were enriched in functions related to cytoskeleton organization development, cellular and organelle organization, and inflammation/immune responses. KLF7 target proteins showed significantly enriched processes related to neutrophil-mediated immunity and granulocyte activation. EGR1 targets were enriched in cytoskeleton organization development and RNA processing, such as mRNA metabolism and RNA catabolic processes. GABPA and YY1 targets were mainly involved in cellular and organelle organization and assembly. The biological processes involved in synaptic functions were enriched for the target proteins MEF2A, SP1, and KLF7. The MEF2A and SP1 target proteins were enriched in the regulation of vesicle-mediated transport, while KLF7 proteins, together with those of SP1, were also enriched in the regulation of intracellular transport. SP4 target proteins were enriched in some biological processes, mainly associated with cellular and organelle organization and assembly functions. In contrast, NRA2A was implicated only in assembly functions.

### 2.3. Altered Pathway Analysis Controlled by Transcriptional Programs in the Cerebellum in Chronic Schizophrenia

**Figure 4:**
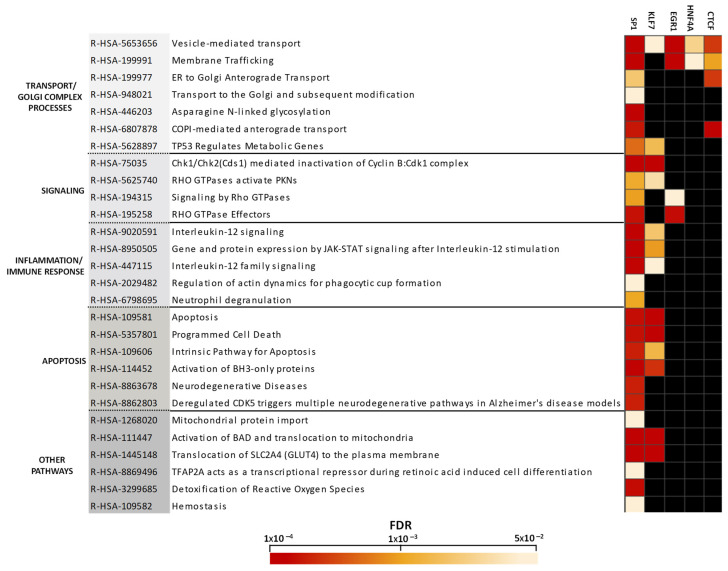
Non-redundant enriched pathways for altered targets of transcription factors. We used the Reactome database for enrichment pathway analysis, and the results are displayed as a heat map created using Perseus software.

Our results revealed pathways significantly enriched (FDR < 0.05) in altered targets of five TFs: SP1, KLF7, EGR1, HNF4A, and CTCF (Figure 4). The enriched pathways were mainly detected in targets regulated by Krüppel superfamily TFs, such as SP1 and KLF7, with 28 and 13 pathways, respectively. SP1 targets showed enrichment in all pathways. The vesicle-mediated transport pathway was under the control of targets of five TFs. EGR1-altered targets were enriched in pathways involved in transport and signaling. HNF4A-altered targets were only enriched in pathways related to vesicle-mediated transport and membrane trafficking pathways. CTCF targets were enriched in pathways involved in transport and processes associated with the Golgi complex. Moreover, SP1- and KLF7-altered targets showed an enrichment in pathways related to signaling, inflammation/immune response, apoptosis, and energy (mitochondrial processes and glucose transport mediated by the translocation of SLC2A4 (GLUT4) to the plasma membrane).

## 3. Discussion

Our study identified 11 potential TFs enriched in the cerebellum in chronic SZ that could control the expression of the 250 significantly altered proteins, contributing to the dysregulation of several biological processes and pathways in SZ. Several studies have implicated 10 out of these 11 TFs in SZ: SP1 [20,21,22], KLF7 and SP4 [23,24,25,26,27,28], EGR1 [29,30,31], HNF4A [32], CTCF [33,34,35], GABPA [33], NRF1 [36,37], NFYA [38], YY1 [34], and MEF2A [39]. Indeed, altered expression in the cerebellum, hippocampus, and prefrontal cortex in SZ has been reported for SP1 and SP4 [15,24]. *EGR1* and *NRF1* mRNA levels have also been shown to be decreased in PFC and cortical tissue, respectively, in SZ [36,40,41]. Together, these results suggest that the alterations in these transcriptional programs are not restricted to the cerebellum and may be present in other brain regions in SZ.

### 3.1. Transcription Factor-Dependently Enriched Biological Processes

#### 3.1.1. Cytoskeleton and Organelle Organization

The enrichment analysis showed that SP1, KLF7, and SP4, which belong to the SP/KLF superfamily, had the greatest number of target genes. The SP/KLF superfamily is characterized by its binding to GC boxes in promoter regions with almost identical affinity due to the high homology in their DNA-binding domains [42]. Our results identified biological processes such as cytoskeleton organization/development, cellular/organelle organization, and pathways related to signaling as the most enriched categories for SP1, SP4, and KLF7. The cytoskeleton mediates a large variety of cellular functions, including supporting cellular morphology and cellular activities such as vesicle trafficking, neuronal migration, and neurite outgrowth [43]. SP1 in astrocytes has been implicated in neurite outgrowth and synaptogenesis [44], while SP4 has been associated with dendritic arborization in the cerebellum [14,45]. KLF7 has been implicated in the enhancement of axon growth [46,47], the formation of dendritic branching in the hippocampus, and altered axon projection in several brain regions [46]. Moreover, KLF7 has been reported to be involved in the maturation of granular neurons in the cerebellum during early postnatal development [46]. In addition, studies performed on the postmortem cerebellum have shown altered levels of SP1 and SP4 proteins linked to negative symptoms in chronic SZ. Altered levels of both transcription factors were also found in the hippocampus in these subjects [15] and in the prefrontal cortex; only SP1 protein levels were reduced in these subjects [24], suggesting the region-specific dysregulation of these TFs in SZ. These reports together with our results point to the possible dysregulation of KLF7 in SZ, leading to the alteration of the maturation of granular cells and axon growth, while the altered expression of SP1 and SP4 could be related to the altered formation of neurites and dendritic arborization patterns. All these processes could eventually lead to altered cell–cell communication in the inner cerebellar circuits and the connection of the cerebellum with other brain regions.

#### 3.1.2. mRNA Processing and Splicing

Our analysis reports that a protein set involved in biological processes related to mRNA processing could be under the transcriptional control of SP1, EGR1, and KLF7, with SP1 target genes being the only ones enriched in splicing. It has recently been shown that alternative splicing could play a role in SZ [48,49]. Many of the archetypal genes associated with SZ, for example, DISC1 [50] and ERBB4 [51], are aberrantly spliced transcripts. However, the molecular mechanism underpinning this aberrant splicing is unknown. A study in mice showed that Sp1 enhanced the transcription of the splicing factor *Slu7*, while the depletion of Sp1 repressed *Slu7* expression, thereby affecting alternative splicing processes [52]. Thus, further studies will be needed to explore the possibility that SP1-dependent altered splicing may mediate the generation of aberrant alternative splicing forms in key genes in SZ physiopathology, such as DISC1 and ERBB4.

#### 3.1.3. Synaptic Function

In our study, the most significant enriched process from synaptic function was vesicle transport linked to MEF2A target genes. MEF2A is a transcription factor expressed in adults and implicated in neuronal development and the formation of postsynaptic granule neuron dendritic claws [53,54]. Moreover, the study of Crisafulli et al. found that at least seven single-nucleotide polymorphisms in MEF2A could be related to SZ [55,56]. Also, MEF2A has been identified as a negative regulator in AMPA receptor expression, which participates in memory processes [57], suggesting that this transcription factor could be involved in cognitive decline in SZ. Therefore, the dysregulation of MEF2A could be responsible for altered synaptic morphology not only in cerebellar granule neurons but also in neurotransmitter vesicle transport to the active presynaptic zone in these neurons in SZ.

In our study, EGR1 target genes were significantly enriched in membrane docking linked to synaptic function but also to signaling processes related to RHO GTPase effectors, which are involved in cytoskeleton organization during vesicle trafficking [58]. Interestingly, different GABA receptor subunits are transcriptional target genes of EGR1 in the hippocampus, which suggest that this transcription factor has a major role in GABA receptor composition, controlling synaptic strength [59]. Indeed, EGR1 has also been widely reported to be a major regulator of synaptic plasticity in different neurons and brain regions, including the cerebellum, in physiological and pathological conditions such as schizophrenia (reviewed in [60]). Thus, our results provide more evidence for the alteration of EGR1 in a pathological context, providing a possible dysregulation of its transcriptional programs involved in synaptic function in SZ in the cerebellum.

### 3.2. Transcription Factor-Dependently Enriched Pathways

#### 3.2.1. Transport and Golgi Complex

Pathways related to transport and the Golgi complex, such as vesicle-mediated and membrane trafficking, were the pathways found to be most enriched for the target proteins of SP1, EGR1, HNF4A, and CTCF. All these pathways are involved in the functioning of the Golgi apparatus. Protein transport from the endoplasmic reticulum to the Golgi complex requires transport vesicles [61]. Recently, it has been proposed that the Golgi phosphoprotein 3 (GOLPH3), which participates in protein trafficking, receptor recycling, and glycosylation in the Golgi, can regulate the transcription of proinflammatory cytokines such as TNF-α; this regulation could be mediated by the EGR1/ERK pathway [62]. This evidence raises the question of whether EGR1 could also be implicated in inflammatory processes in SZ. Moreover, all the TFs involved in the transport and the Golgi complex, such as SP1 [15,24], EGR1 [63], HNF4A [32], and CTCF [33], have been previously reported to be altered in SZ [63]. However, the role of these TFs in anterograde transport or functions associated with the Golgi apparatus in the context of SZ is unknown.

#### 3.2.2. Immune Response and Inflammatory Processes

Although the neurodevelopmental hypothesis is well accepted, the inflammation and dysregulation of immune mechanisms and degenerative views have also been suggested as hypotheses, which has generated significant debate in the field [64,65,66,67,68,69,70,71,72]. An imbalance in the levels of proinflammatory and anti-inflammatory cytokines has been related to symptoms and cognitive decline in SZ [73,74]. In our study, biological processes and pathways related to the immune response were found to be enriched, linked to specific transcriptional programs. The transcriptional control of the targets involved in inflammatory events could be regulated by some members of the Krüppel-like factor family, such as SP1 and KLF7. KLF7 has been related to increases in the levels of IL-6, which play a role in both inflammatory and anti-inflammatory responses [75]. KLF7 could promote an increase in IL-6 through PKCζ/NF-κB [76] and TLR4/NF-κB/IL-6 signaling [77]. In addition, studies have reported high levels of IL-6 in SZ subjects [78,79]. A study reported that KLF7 could induce macrophage activation [76,77]. Moreover, several members of the Krüppel-like factor family, such as KLF2, KLF4, and KLF6, have been reported to be involved in the immune system and inflammation [80,81,82], which is in line with our results. Thus, taken together, these findings suggest that KLF7 could have a relevant role in inflammatory processes in SZ.

Another member of the Krüppel-like factor family is SP1. SP1 has been associated with the activation of interleukin 21 receptors in T cells [83,84], which mediate the activation of several cell types involved in the immune response [85]. Furthermore, SP1 has been implicated in interleukin 12 (*IL-12*) expression [86]. IL-12 induces the differentiation of T-helper 1 cells [87] during the adaptive immune response. In this sense, altered IL-12 levels have been reported in the plasma of SZ subjects [88,89]. Also, SP1 induces the activation of macrophage inflammatory protein-2 (*MIP-2*), which is involved in recruiting neutrophils to inflammatory regions [90]. In addition, SP1 has also been implicated in the crosstalk between the interferon regulatory factors and NFκB pathways, thereby contributing to the TLR-dependent antiviral response [91]. In SZ, it has been reported that SP1 could interact with the TLR4-MyD88-IκBα-NFκB pathway, which mediates its interaction with NFκB [92]. Thus, SP1 could be an activator of the immune response. The dysregulation of *IL-12* expression due to the altered function of SP1 could lead to the dysfunctional differentiation of T-helper cells and an altered adaptive immune response in SZ. Thus, our study suggests the possible participation of SP1 in inflammatory processes in SZ subjects.

#### 3.2.3. Apoptotic Events

Disseminated apoptotic events in the CNS throughout the developmental period and later phases impact the emergence of SZ and the progression of the disease [93,94]. These apoptotic processes support the neurodegenerative hypothesis proposed for SZ [95,96]. However, the transcriptional program involved in this process is unknown. Our analysis revealed that SP1 and KLF7 could participate in mitochondrial apoptosis. While some studies have demonstrated that the overexpression of *SP1* could induce apoptosis, others have reported that the depletion of *SP1* increases the sensitivity of cells to DNA damage [97,98,99] and eventually leads to apoptosis. Thus, *SP1* could have a dual function in apoptosis. Moreover, it has been reported that the depletion of *KLF7* increases cell apoptosis in animal models [100]. Although KLF6 has been reported to be a regulator of mitochondrial function during apoptosis [101,102], no information is available for KLF7 regarding this function. However, it has recently been proposed that KLF7 could inhibit inflammatory and apoptotic processes in cell lines via NRF1/KLF7 [103]. Thus, in the context of SZ, the altered expression of *SP1* and *KLF7* could activate apoptotic signaling pathways in the CNS and contribute to the disseminated apoptosis described in SZ [104].

#### 3.2.4. Limitations of the Study

Several limitations are identified in this study based on the human postmortem brain to understand the transcriptional programs altered in chronic schizophrenia. Firstly, patients with elderly chronic schizophrenia had been taking long-term and heterogeneous antipsychotics medications. A study has shown that long-term haloperidol doses induce the dysregulation of cytoskeleton proteins and spine-related proteins in dopaminergic areas in the cortex cerebral, which could influence vesicular transport and synaptic activity [105]. Thus, advanced age, the long duration of the illness, and antipsychotics could have influenced the transcriptional programs and the molecular pathways described in this study. Secondly, our study cohort constituted only men. Further studies are needed to also explore these transcriptional programs in women.

## 4. Materials and Methods

### 4.1. Postmortem Human Brain Tissue

Tissue samples were from gray matter obtained from the cerebellar lateral cortex and belonged to a cohort of subjects with chronic schizophrenia (*n* = 12) and healthy controls (*n* = 14) previously described [19]. Briefly, these samples were obtained from the neurologic tissue collection of the Parc Sanitari Sant Joan de Déu Brain Bank (Barcelona, Spain and the Institute of Neuropathology of the Universitari de Bellvitge Hospital (Barcelona, Spain), respectively. Clinical and tissue-related features are detailed in Table S1.

### 4.2. Bioinformatic Analysis

To identify transcription factor enrichment, we used FunRich Tool v.3.1.3. To represent the results obtained with FunRich Tool, we used Graph Prism version 7.00 (GraphPad Software, San Diego, CA, USA). To perform non-redundant enriched category analysis for Gene Ontology and pathways, we used Webgestalt (WEB-based Gene SeT Analysis Toolking) (https://2019.webgestalt.org/#, Data sources for WebGestalt 2019 was updated on 14 January 2019) and the method of Over-Representation Analysis (ORA), supported by Fisher’s exact test [106]. For pathway analysis, we used the Reactome database. The enrichment analyses were set to FDR = 0.1. To represent the enrichment analysis, we created a heat map with the Perseus software platform (version 1.6.1.3. https://maxquant.net/perseus/, 8 April 2025).

## 5. Conclusions

The altered proteins in the cerebellum in schizophrenia include the target genes of only 11 transcription factors: SP1, SP4, EGR1, KLF7, HNF4A, CTCF, MEF2A, GABPA, NRF1, YY1, and NYFA. Our results show that transport-related pathways are enriched for SP1-, KLF7-, EGR1-, HNF4A-, and CTCF-altered targets. Signaling-related pathways are enriched for SP1-, KLF7-, and EGR1-altered targets. SP1 and KLF7 could contribute to the signaling dysfunction induced by dendritic arborization alterations and to the loss of the maturation of granular cells in the cerebellum, respectively. Pathways involving inflammatory/immune responses and apoptosis are enriched with SP1- and KLF7-altered targets. SP1 could participate in the immune response and induce the differentiation of T helper cells, and KLF7 could induce macrophage activation. This suggests that SP1 and KLF7 could play a prominent role in the cerebellum in chronic schizophrenia. Together, all these findings suggest that the altered function of a limited number of transcription factors could have an impact on disseminated pathways involved in different cellular functions.

## Data Availability

The original contributions presented in this study are included in the article/Supplementary Materials. Further inquiries can be directed to the corresponding author.

## Supplementary Materials

The following supporting information can be downloaded at: https://www.mdpi.com/article/10.3390/ijms26083653/s1. Reference [107] is cited in Supplementary Materials.

## Abbreviations

SZ	Schizophrenia
CB	Cerebellum
CCTC	Cortico-thalamo-cerebellar circuit
CNS	Central nervous system
TFs	Transcription factors
NKX2-1	Homeobox protein Nkx-2.1
SP1	Transcription factor Specificity Protein 1
SP4	Transcription factor Specificity Protein 4
KLF7	Krüppel-like factor 7
EGR1	Early growth response protein 1
HNF4A	Hepatocyte nuclear factor 4-alpha
CTCF	Transcriptional repressor CTCFL
GABPA	GA-binding protein alpha chain
NRF1	Endoplasmic reticulum membrane sensor NFE2L1
NFYA	Nuclear transcription factor Y subunit alpha
MEF2A	Myocyte-specific enhancer factor 2A
YY1	Transcriptional repressor protein YY1
